# Management of Psychosis in Parkinson's Disease: Emphasizing Clinical Subtypes and Pathophysiological Mechanisms of the Condition

**DOI:** 10.1155/2017/3256542

**Published:** 2017-09-12

**Authors:** Raquel N. Taddei, Seyda Cankaya, Sandeep Dhaliwal, K. Ray Chaudhuri

**Affiliations:** Maurice Wohl Clinical Neuroscience Institute and NIHR Biomedical Research Centre, Institute of Psychiatry, Psychology and Neuroscience, King's College Hospital, London, UK

## Abstract

Investigation into neuropsychiatric symptoms in Parkinson's disease (PD) is sparse and current drug development is mainly focused on the motor aspect of PD. The tight association of psychosis with an impaired quality of life in PD, together with an important underreporting of this comorbid condition, contributes to its actual insufficient assessment and management. Furthermore, the withdrawal from access to readily available treatment interventions is unacceptable and has an impact on PD prognosis. Despite its impact, to date no standardized guidelines to the adequate management of PD psychosis are available and they are therefore highly needed. Readily available knowledge on distinct clinical features as well as early biomarkers of psychosis in PD justifies the potential for its timely diagnosis and for early intervention strategies. Also, its specific characterisation opens up the possibility of further understanding the underlying pathophysiological mechanisms giving rise to more targeted therapeutic developments in the nearer future. A literature review on the most recent knowledge with special focus on specific clinical subtypes and pathophysiological mechanisms will not only contribute to an up to date practical approach of this condition for the health care providers, but furthermore open up new ideas for research in the near future.

## 1. Introduction

Nonmotor symptoms have an important impact on quality of life in PD patients and their caregivers and are largely recognized as such by a growing number of health care providers [1, 2]. Psychosis is recognized as one of the most frequent and disabling nonmotor symptoms in PD with prevalences of 20% up to 70% in advanced stages of the condition [3]. Its relevance is such that it has even been named as the main feature of one of the seven proposed nonmotor subtypes of PD described by Sauerbier et al. [4]. In this review we aim at providing an up to date practical approach to psychosis in PD, with especial emphasis on clinical subtypes and pathophysiological mechanisms underlying this condition with the aim of leading to better intervention strategies in the nearer future.

## 2. Defining PD Psychosis

### 2.1. History

The history of psychosis in PD goes back to the early 19th century, where the presence of mental disturbances among PD patients was described as being rare and was accounted for as either a consequence of a chronic disease evolution or regarded as coincidental [5]. After an outbreak of encephalitis lethargica between 1915 and 1926, a condition of unknown origin with acute onset and often chronic persistence of various neurological symptoms, including headache, lethargy, catatonia, parkinsonism, and tremor, a potential link between an altered mental state and parkinsonism was proposed and the first idea of complex psychotic symptoms in postencephalitic parkinsonism (PEP) cases was described [6, 7]. In more recent years however, the etiologic relationship between the encephalitis outbreak and the alleged PEP has been discussed as controversial due to a lack of consistency in clinical features and in the onset of symptoms and the possibility of other causes of parkinsonism has been postulated [7, 8]. Moreover, in subsequent years, confusional states were reported under treatment with L-Dopa and later under dopamine agonist therapy in PD patients, giving rise to this new core feature in PD. In 1995 the first review on drug-induced psychosis in PD was published by Factor et al., leading to the first international awareness of this PD complication [9].

Currently, under various searching terms on psychotic symptoms in PD, including the terms hallucinations, psychotic symptoms, illusions, delusions, and misperceptions among others, over 4000 articles and reviews can be found, dated back as far as 1945 in the current literature (PubMed), being the first description found in a book published in 1921.

### 2.2. General Psychosis and PD Psychosis

With regard to the clinical definition of the main features of psychosis, which include hallucinations, illusions, and delusions, current ICD-10 guidelines define hallucinations as a disorder characterised by a false sensory perception in the absence of an external stimulus, whereas an illusion is regarded as a misperception of an externally present stimulus. In contrast to classical hallucinations and illusions, delusions are a false interpretation of the experienced misperceptions, often involving topics of persecution, imposters, or grandiosity. Some specific forms of delusions such as the Cotard syndrome (implying nihilistic delusions, hypochondriacal delusions, and delusions of immortality) [10–12], Capgras syndrome (including having the conviction that a family member or friend has been replaced by another), and Othello syndrome (being described as a delusional jealousy) have further been named [13, 14], the latter showing an association with dopamine agonist therapy and an improvement after its reduction. The current diagnostic criteria from ICD-10 based guidelines for acute and transient psychosis are shown in Box 1; other definitions of psychosis falling under the term of schizoaffective disorders will not be further developed in this review.

With regard to psychosis arising in PD patients, it is defined as the occurrence of hallucinations, delusions, or both, most of the psychotic symptoms being of visual character, with the potential of other sensory modalities to be involved as well [16]. They can be subdivided into minor and nonminor hallucinations [17] and minor symptoms can be subdivided into three forms, known as illusions (meaning that real objects are seen transformed into other shapes/figures), passage hallucinations (implying, e.g., hallucinated objects or dots of light passing in the peripheral visual field), or presence hallucinations (including the sense of a nearby person or animal) [18, 19].

As to the clinical presentation of the psychotic features in PD, they are mostly of visual character, including complex perceptions containing animals, persons, or objects, and are normally not frightening to the patients, who clearly recognize them as being abnormal [17, 20]. Most occur in poor light conditions and reduced stimulus environment, more often at night, and last for seconds to minutes [17]. The visual hallucination might be in black and white or in colour and is perceived by most of the patients as “bothersome.” A common form of visual hallucination is the perception of bugs on the walls or on the floor. Later occurring auditory hallucinations are mostly described as indistinct sounds (e.g., a radio playing in the room, a band performing on the street, or a conversation taking place outside the room) and are therefore distinct to the symptoms of psychosis in schizophrenia patients, where a threatening or denigrating character is typical.

In early PD stages, hallucinations typically occur with preserved insight, whereas in later disease stages this characteristic is lost and patients might not recognize the misperceptions as unreal anymore, giving rise to the above-mentioned term of (secondary) delusions [17]. When other sensory modalities are involved, auditory but also more rarely olfactory or tactile hallucinations may occur [21]. Proposed diagnostic criteria for psychosis in PD arose from a consensus conference in 2007 by the NONDS/NIMH work group [22] and are shown in Box 2.

The prevalence of PD psychosis varies widely; among untreated PD patients it is reported to occur “rarely” [24], whereas in treated patients prevalence widely differs in the literature, with reported illusions or hallucinations occurring in 15–40% of treated PD patients [25] and an estimated development of psychosis in up to 60% of PD patients after a 12-year disease duration [26] or even 70% in “advanced” PD stages [3]. The occurrence of minor phenomena is reported to range between 17% and 72% in the current literature [27–30]. A recently published study on 423 drug-naïve PD patients followed up for 3-4 years showed an overall prevalence of psychotic symptoms in 27% of the PD patients after a median time of 19 months [19].  Table 1 shows the different prevalence rates of PD psychosis reported among various studies.

Various risk factors have been linked with the development of PD psychosis, such as the use of dopaminergic drugs as one of the first described ones. A recent study by the Parkinson's Progression Markers Initiative (PPMS) found that there were no significant differences with regard to occurrence of PD psychosis after starting L-Dopa or dopamine agonists, but a higher proportion of PD psychosis was observed over time after a period of 24 months in PD patients treated with dopamine agonists compared to the ones treated with L-Dopa [19].

Nowadays, emphasis on various other related factors has been attributed to leading to psychosis in PD patients, including higher age, later disease onset, higher PD severity (H&Y state), longer PD duration [27, 32], hyposmia [33], cognitive impairment, depression [34], diurnal somnolence, REM sleep behaviour disorder [28], visual disorders, severe axial impairment, autonomic dysfunction, and high medical comorbidity and polypharmacy, especially including the use of psychoactive drugs [3, 23, 35].

To assess the risk of developing psychosis, several scales are accessible either to address the presence of psychosis or to establish its severity. These include the PD nonmotor symptom scale developed by Chaudhuri et al. [36] and subscores of the MDS-UPDRS scale [37], as well as more specific scales directed towards assessing specifically psychiatric comorbidity in PD, including the Parkinson Psychosis Questionnaire [38], the Scale for Assessment of Positive Symptoms [39], and the Scale for Evaluation of Neuropsychiatric Disorders in PD [40]. These available scales are summarised in Table 2.

### 2.3. Other Psychotic Syndromes

In the following section, we will revise three specific subtypes of psychosis that may cooccur or contribute to the “classical” PD psychosis described above, in order to provide a complete revision on this topic.

#### 2.3.1. Charles-Bonnet Syndrome

This syndrome with an estimated prevalence of 0.4% up to 30% in the overall population [41–45] dates back to a Swiss scientist named Charles-Bonnet, who described the occurrence of detailed visual hallucinations including figures, persons, and animals in his visually impaired grandfather, after losing his sight due to a bilateral cataract surgery. But it was not until 1967 that another Swiss scientist called G. de Morsier described this syndrome with the currently used term Charles-Bonnet syndrome. Diagnostic criteria for this syndrome are yet a topic of controversy, the current definition being the presence of visual hallucinations occurring as a result of ocular or visual pathway disease. The visual hallucinations can be of simple or complex nature, including hallucinations of faces or people-like figures [46], and normally last for seconds to a few hours. Most patients do not describe a negative or fearful experience during their visual hallucinations [41] and partial or full insight of their unreal character, absence of coexisting psychological disorders, and preserved intellectual capacity is typically observed [47]. This condition has been described at different ages with no specific age dependent prevalence.

In PD patients, the loss of dopaminergic neurons in the brainstem leading to a deficiency of dopamine has been linked with an impairment in visual pathways, involving mainly the retina, as well as central pathways [48]. Whether the presence of visual hallucinations in PD patients could be at least partially explained by a visual impairment due to dopaminergic loss in form of a Charles-Bonnet syndrome needs to be further elucidated. Currently, impaired vision has been associated with the occurrence of visual hallucinations in PD patients as described above [49].

#### 2.3.2. “Malignant”: DLB/PD Dementia with Psychosis

Dementia with Lewy bodies (DLB) and PD associated dementia (PDD) are two separate entities, yet both involving a similar pathological pathway of deposition of Alpha-synuclein within the brain in form of Lewy bodies. Presence of hallucinations is a common hallmark in both entities, being described in as many as 25–30% of DLB and PDD patients [22] and being most commonly of visual character, although also acoustic and haptic (tactile) hallucinations can occur [46]. In the progress of more severe presence of cognitive decline, visual hallucinations tend to shift from a blurred character among PD and PDD patients to fully formed complex visual hallucinations among DLB patients [46]. The distinction between both types of dementia is nonetheless a challenge and often complicated by an overlap of the clinical presentations and an unclear time window. DLB is commonly diagnosed, when cognitive impairment occurs within a year of development of parkinsonian symptoms, whereas PDD is defined as dementia occurring at least 1 year after motor symptom onset [50]. A recent study by Fritz et al. could additionally find some clinical features that differ between DLB and PD which included a slower speed, shorter stride length, and increased stance phases of gait as well as a higher frequency of falls among DLB when compared to PD patients [51]. The differentiation and correct recognition thus pose a challenge to the clinician.

With regard to the presence of hallucinations, in DLB these tend to occur early in the disease course and are not associated with dopaminergic medication, whereas in PDD they tend to develop in later stages and to be related to intake of dopaminergic therapy, mostly of dopamine agonists [52]. Autopsy series in DLB with hallucinations showed a deposition of Lewy bodies in the inferior temporal cortex, with similar anatomical correlates found in autopsies of PD patients with hallucinations, so that hallucinations were correlated with the presence of Lewy body pathology in the temporal lobes [53]. Nonetheless, a more recent study assessing structural changes in dementia by means of MRI scans could correlate the presence of hallucinations with a cortical atrophy in visual pathways, rather than an anatomical correlate in temporal regions [54]. As a potential further biomarker of hallucinations in PDD, a study on the effect of fluctuating cognition in DLB and PDD showed a significantly higher prevalence of hallucinations among DLB and PDD patients when fluctuating cognition features were present [55].

#### 2.3.3. Acute Psychosis in PD Patients

Psychosis normally develops in the course of PD, gradually increasing in severity over time. If isolated visual hallucinations manifest independently or before the onset of “classical” PD psychosis, which is defined as lasting for over 1 month, it has been described as mostly resulting from medication [56]. An acute setting with sudden onset of psychotic symptoms must be regarded as an emergency situation [57]. Apart from recent changes in PD medication and acute intoxications associated with dehydration or other metabolic disorders, less frequent differential diagnosis such as cerebral infarction, intracranial haemorrhage, or CNS infections needs to be addressed. It is well known that some specific medical comorbidities can acutely trigger a psychotic episode or influence the severity of its symptoms [3], including infections, dehydration, sleep deprivation, irregular nutrition, psychosocial stress, deprivation or overload of sensory inputs, operations, metabolic alterations, dopaminergic drugs, and some other not antiparkinsonian drugs such as beta-blockers or corticosteroids. Under benzodiazepines a paradoxical reaction with restlessness, excitation, and euphoria may occur.

Another associated acute neuropsychiatric disturbance in PD is delirium, which implies a fluctuating alteration of attention accompanied by an additional disturbance in cognition, as defined by the DSM-V criteria [58] and has been considered as an acute decompensation occurring in ageing and/or dementia, particularly among vulnerable brains such as PD affected individuals. The estimated prevalence is of 8 to 24% [59–61] and a 5 times higher risk of developing delirium among PD patients when compared to healthy controls has been described in a recent study by Lubomski et al. [59]. A differentiation of delirium and psychosis, although challenging and not thoroughly studied in the current literature, can be considered with the features in Table 3.

A further acutely occurring alteration of mental status is the acute confusional state, which overlaps with delirium and has to date no condensed distinctive features from it. In fact the terms acute confusional state and encephalopathy are commonly used synonymously with the term delirium. While the symptom confusion itself refers more specifically to an inability to think coherently and an overall depressed sensorium, it poses an essential feature of the above defined term delirium [62, 63].

Another acutely occurring complication in PD is acute akinesia or akinetic crisis, which primarily involves worsening of motor performance lasting for ≥48 hours despite treatment [64] but is frequently associated with psychotic and cognitive symptoms as well [65]. It poses a life-threatening but rare complication in PD, having an estimated prevalence of 0.3% and mainly arising secondary to infections, surgical interventions, or changes in treatment [66]. The main clinical features imply a severe rigidity and akinesia associated with hyperthermia, dysautonomia, dysphagia, and/or increased serum muscle enzymes levels. Although frequently severe and associated with a high mortality rate of around 15%, also less severe forms known as “forme frusta” have been described [67, 68]. The clinical similarities to the malignant neuroleptic syndrome led this condition to also be named as neuroleptic malignant-like syndrome, malignant syndrome, or parkinsonism-hyperpyrexia [67], although some of these terms might be misleading, since an independent occurrence from neuroleptic drug use has been reported [64]. A study specifically addressing the adverse events arising from antipsychotic use in PD patients found an overall highest incidence of adverse reactions under quetiapine therapy, followed by clozapine and olanzapine but when addressing the occurrence of the adverse event “neuroleptic malignant syndrome,” quetiapine was the most frequently concomitantly given medication [69], which goes along other published case reports on the association of quetiapine and the occurrence of neuroleptic malignant syndrome in PD [70].

## 3. Pathophysiology and Potential Biomarkers of Psychosis in PD

### 3.1. Introduction

Psychosis in PD patients is mostly arising in patients with a clear sensorium in a chronic setting after a long disease duration and is triggered or enhanced by pharmacological factors. Nonetheless, other recent risk factors have been studied, giving rise to a potential multifactorial underlying aetiology and thus physiopathology. The differentiation between early and late onset psychosis in general, and also specifically in PD, is not currently validated, having some studies randomly set cut-off values at 2–4 years of PD onset to determine early versus late onset psychosis [71]. More interestingly, the assessment of psychosis as a potential prodromal feature could give rise to finding biomarkers in PD, in order to predict its occurrence and treat it at early stages. In this section we will review the pathophysiology of psychosis, focusing on prodromal/premotor psychosis and on psychosis arising in the course of PD (with no differentiation between early and late onset), and then address potential biomarkers of PD psychosis.

### 3.2. Premotor versus Late Occurring PD Psychosis

To our knowledge only one study has been performed and recently published to analyze the presence of psychotic symptoms as a feature of the premotor state of PD; Pagonabarraga et al. studied a cohort of 50 drug-naïve PD patients and compared them with 100 healthy controls to assess the presence of hallucinations [72]. They found an overall prevalence of minor hallucinations in the untreated PD group of 42%, the onset of these being 7 months to 8 years prior to motor symptom onset. The prevalence of hallucinations in the control group was 5%. When comparing the cohort of PD patients and healthy controls, the groups did not differ in baseline characteristics, apart from a significant impairment in global cognitive function in the PD group compared with the control group. Nonetheless, dementia criteria were not met in any of the subjects included in the study. When then comparing the PD patients with and without hallucinations, older age and the presence of rapid-eye-movement behaviour disorder (RBD) were statistically significantly correlated (*P* < 0.05) as seen in Table 4. This preliminary study sheds light on a potential prodromal occurrence of PD psychosis and proposes risk factors that could help recognize at-risk PD patients. Further studies with wider sample sizes are nonetheless needed.

As mentioned previously, the prevalence of psychosis in the course of PD varies widely in the literature. Psychosis itself represents a relevant burden in PD, involving their caregivers and health professionals, since it has been associated with a higher morbidity and mortality [73]. Additionally, together with depression and dementia, it poses one of the most prevalent nonmotor features in PD [74], accounting for up to one-fifth of the complications arising over time [75].

As to potential biomarkers, a strong correlation of psychosis with depression and REM behaviour disorders has been described by Lee and Weintraub in a study on 191 nondemented PD patients [74], where the risk of developing psychosis with comorbid depression and sleep related disorders was 5 times higher. In this study they identified an overall prevalence for psychotic symptoms in 21% of PD patients. The variables associated with the occurrence of psychosis were similar to previous studies, Hoehn and Yahr (H&Y) stage, disease duration, Unified PD Rating Scale (UPDRS) motor score, depression, anxiety, RBD symptoms, daytime sleepiness, and apathy. A nonstatistically significant trend towards psychosis under higher L-Dopa dosages was found, but no correlation with dopamine agonist therapy could be established, in contrast to previous studies. A lower Mini Mental State Examination (MMSE) score also indicated a trend towards psychosis.

The risk factors of psychosis thus seem to be related to multiple pathways involving different neurotransmitter systems, complicating the understanding of the underlying processes taking place.

### 3.3. Pathophysiology of PD Psychosis

The pathophysiological processes underlying PD psychosis can be subdivided into intrinsic (neurotransmitter-dysfunction related and thus not externally induced) and extrinsic (drug-related and thus a direct result of the use of pharmacological agents). While intrinsic PD psychosis is thought to be caused by alterations in dopamine, serotonin, and acetylcholine systems involving subcortical projections as well as synaptic and neuronal changes in limbic and cortical structures [76], extrinsic PD mainly involves dopaminergic or anticholinergic therapies, especially dopamine agonists [35, 72].

To better understand the neurotransmitter dysfunctions underlying the development of hallucinations, the effect of hallucinogenic agents with known mechanisms of actions is of advantage. Classically, hallucinogenic agents were subdivided into those affecting the cholinergic system and those involving the aminergic system, herein dopaminergic and serotoninergic agents being included [77]. The described clinical characteristics and associated hallucinations caused by these two distinct systems also differ: while the effects caused by anticholinergic agents are associated with peripheral autonomic features, confusion, disorientation, and visual hallucinations, mostly poorly formed and of a threatening nature, the symptoms caused by aminergic agents are characterised by a heightened awareness of objects, forms, and colours with a clear sensorium, sometimes involving the presence of hypnagogic phenomena of a dream-like quality [77].

A novel model to clarify the underlying mechanism of psychosis in PD proposed by Wolters [78] suggests that the DA stimulated orbitofrontal output activates dorsal raphe neurons, which release serotonin and activate 5HT2a receptors. These receptors stimulate GABAergic neurons which influence dopamine neurons in the ventral tegmental area [79] through the neurotransmitter glutamate. Finally excitation of the limbic system and inhibition of the prefrontal cortex take place, giving rise to an impaired selection and weighing of external environmental stimuli, thus leading to a mis/overinterpretation of external inputs.

Other hypotheses come from a combination of neurotransmitters from Birkmayer and Riederer [80], shedding light on a dopamine/serotonin imbalance as an underlying mechanism of psychosis. This goes along postmortem studies, which found a loss of serotonin among PD patients [81] and the evidence on the positive effect of pharmacological agents which act by activating the 5-HT2 receptors, such as pimavanserin.

Since cholinergic deficiency is present not only in Alzheimer's disease, but also in PDD and DLB [82], and there is a close link between cognitive impairment and psychosis, a potential implication of this neurotransmitter could be considered. This led to a further hypothesis on the pathophysiology of psychosis by Perry et al., who proposes a cholinergic/serotoninergic imbalance [83, 84]. Cholinergic depletion is already regarded as a potential underlying cause of psychosis in DLB alongside visuospatial processing deficits, which could therefore be thought of having a similar mechanism in PD psychosis.

**Figure 1:**
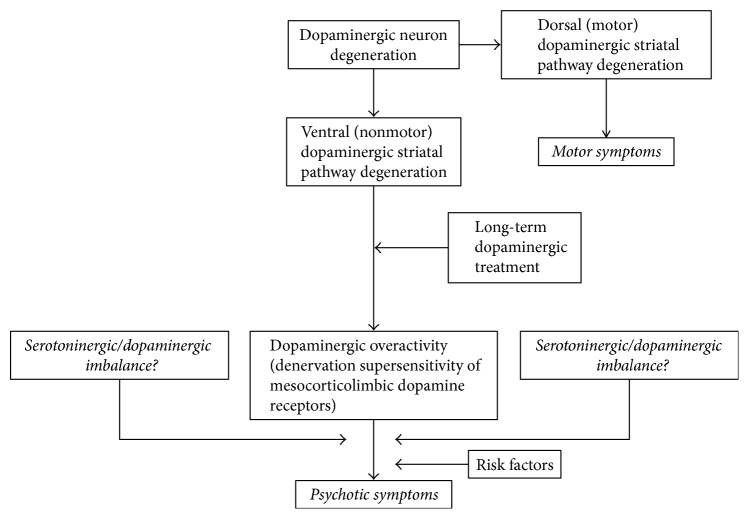
Proposed neurochemical pathways in the pathogenesis of psychosis in PD. DA, dopamine. Reference: Papapetropoulos et al., 2005 [31].

Papapetropoulos and Mash propose, based on the above-mentioned findings, a neurochemical pathway for PD psychosis pathogenesis [31], with a disruption in mesolimbic dopaminergic pathways that lead to a supersensitivity of the implicated DA neurons in the striatum along with facilitating systems involving the serotoninergic/dopaminergic and serotoninergic/cholinergic balance as seen in Figure 1. Other factors, such as genetic variances or environmental factors, were also postulated in this study; studies on potential DA receptor genes variants implicated in hallucinations in PD have nonetheless shown controversial results, 2 studies suggesting an influence on psychosis by some genes [85, 86], but one other study not being conclusive on this relationship [87].

It is well known that treatment with antiparkinsonian drugs may induce psychotic features in PD patients, posing a limitation in their application and even leading to their discontinuation if severe symptoms arise. Dopamine replacement therapy is the current mainstay treatment of PD but its pulsatile administration leads to an increase in both tonic and phasic dopamine signalling [88, 89]. While the externally administered replacement of dopamine in areas of dopaminergic cell loss is of beneficial effect, the stimulation of relatively unaffected areas such as the ventral tegmental area and the ventral striatum can impair the functioning of these areas.

In a study conducted on PD patients with marked hallucinations under antiparkinsonian treatment, a clear sensorium even after long-term anticholinergic treatment and a precipitation of psychosis after increases of dopaminergic or anticholinergic drugs with a typically similar appearance of hallucinations within each patient could be found, whereas decreases in the dosage of dopaminergic or anticholinergic treatment showed to improve hallucinations [90]. These findings support a pathophysiological mechanism directly related to changes in both dopamine and acetylcholine levels within the brain related to externally administered medication. Animal models on the effect of long-term L-Dopa therapy have also shown changes at dopamine receptor sites along behavioural changes. Particularly mesolimbic areas, with a known high density of dopaminergic and cholinergic nerve terminals, have been implicated in the pathophysiology of these underlying neuronal changes [90]. Furthermore, animal studies on MPTP-treated monkeys showed upregulations of D1 and D2 receptors in the denervated striatum [91], which could be replicated in humans in further studies, showing a potential compensatory upregulation of DA receptors presumably due to nigrostriatal DA denervation (so-called denervation supersensitivity) [92, 93].

Interestingly, abnormal dopaminergic transmission has also been observed in schizophrenia and schizotypal trails and has been considered a main characteristic of its underlying pathophysiology [94]. In PD patients receiving dopaminergic therapy, some positive schizotypal trails could be found, postulating a potentially, at least partially common pathway of psychosis development [95, 96]. Externally administered dopamine therapy is thought to stimulate supersensitive striatal and mesolimbic dopamine receptors thus leading to the generation of visual hallucinations, the connections to frontal regions being specifically implicated and thus an anatomically/topologically comprehensive pathway for the induction of PD psychosis [77]. Nonetheless, a small study on intravenous L-Dopa infusions in 5 nondemented PD patients with daily hallucinations did not trigger visual hallucinations, postulating that high levels of L-Dopa and thus DA receptor activation alone do not cause visual hallucinations solely, so that more complex systems might be involved [97].

The link between cognitive impairment and PD psychosis has been thoroughly described in the literature [74, 98] with a recent review stating a correlation of PD psychosis in patients with impairment in mainly cognitive executive, attentional, and visuospatial domains [98]. Further, PD psychosis has also been described as a risk factor for the development of PD dementia [32, 74], giving rise to a potential link between both complications. Accordingly, a recent study by Factor et al. [99] confirmed an association between hallucinations in PD and global cognitive decline but also described for the first time a lack of correlation between delusions and cognition in PD patients. Structural neuroimaging studies have shown evidence of pronounced atrophies in frontal and limbic areas, as well as in the visual pathways and cortex in PD patients with hallucinations and cognitive impairment [100], although some controversy within the results has also been reported [101]. With regard to neurotransmitter systems implied in the pathophysiology of both conditions, cholinergic and dopaminergic pathways are thought to be involved [102]. Evidence in favour of an underlying cholinergic degeneration in PD psychosis among cognitively impaired patients has been supported by the fact that choline acetyl transferase reduction has been found in the neocortex of hallucinating PD as well as Lewy body dementia patients [103, 104] and the fact that cholinesterase inhibitors improve psychotic symptoms in some cases of Lewy body dementia patients [105, 106]. With regard to the dopaminergic system, the finding of a missing relation between delusions and cognitive decline [107, 108], the reported cases were found to be described in PD patients on dopamine agonists and the symptoms as reversible after dopaminergic drug discontinuation, shedding light on a direct and strong involvement of the dopaminergic pathways in the development of psychotic features defined as delusions. The complexity and controversy among clinical presentations, imaging findings, and neurotransmitter imbalance thought to be involved [18] have been recently addressed in a thorough review and have shed light on the need of future research into this field.

### 3.4. Potential Biomarkers of PD Psychosis

A biomarker is defined as a characteristic that can be objectively measured and that can indicate a normal biological process, a pathogenic process, or a pharmacologic response to a specific therapy [109]. Biomarkers can range from clinical, neuroimaging, and biochemical to genetic or proteomic characteristics and their purpose can be to confirm a diagnosis, serve for epidemiological screening, predict an outcome, monitor disease progression, or assess and predict response to a treatment. In the thorough search for a therapy for PD in the past 30 years, next to the complexity of the disease itself, the lack of reliable tools available to monitor progression and to observe the effects of the interventions has been a major drawback. Screening for biomarkers in PD is therefore highly relevant, but no reliable ones are readily available [110]. To this purpose, the PPMI (Parkinson Progression Marker Initiative) is currently undergoing an observational, multicentre, international study designed to evaluate potential biomarkers of PD progression comprising 400 recently diagnosed PD patients and 200 healthy subjects among a total of 21 centres [111].

Apart from the above-mentioned associated risk factors in PD, specific biomarkers for PD psychosis have to our knowledge not been studied so far; nonetheless biomarkers for cognitive decline, which is a risk factor for the development of psychosis in PD, have been recently published. In a study by Skogseth et al. [112], cerebrospinal fluid (CSF) parameters and cognition in PD were assessed, finding a significant correlation between reduced Alpha-synuclein and reduced composite cognition and executive-attention domain scores. Associations between T-Tau and A-beta42 were not significantly associated with PD-MCI. Alongside this finding, another recent study by Stav et al. showed a significant correlation between A-beta38, A-beta40, and also Alpha-synuclein in PD patients with MCI [113]. In the current literature on biomarkers in cognitive decline in PD, the most consistent finding is an association of reduced A-Beta 42 in CSF, while the findings on T-/P-Tau are inconsistent [114]. With regard to biomarkers for psychosis (in non-PD subjects), a recent study on leukocytic miRNA comparing healthy subjects with persons who were at high risk of psychosis without progressing to psychosis and individuals who did develop psychosis over a course of 2 years showed a specific pattern of expression of small regulatory miRNAs in people who developed psychosis compared to those who did not [115]. They could not find individual miRNAs with statistically significant power; nonetheless a sum of 5 miRNAs was proposed to indicate a progression towards psychosis. This goes along another study by Gardiner et al., in which miRNAs were found to be downregulated in 112 schizophrenia patients when compared to 76 healthy controls [116]. Whether these findings could be extrapolated to PD psychosis remains open and needs further studies.

Along this biochemical biomarkers, imaging studies in schizoid and schizotypal personality disorders have found greater volume loss in the superior part of the corona radiata [117] as well as smaller neocortical grey matter volumes with larger sulcal CSF relative volumes [118], as a potential further biomarker of psychotic trails in schizotypal individuals compared to healthy controls. As in the case of the mentioned biochemical biomarkers, further studies are needed with regard to imaging biomarkers as well.

## 4. Management of PD Psychosis

### 4.1. Nonpharmacological Treatment

The importance of a multidisciplinary approach for the treatment of PD psychosis has been revised by several authors in the literature and implies the involvement of psychiatrists and other mental health professionals, neurologists, and functional neurosurgeons [119]. With regard to noninterventional treatments, psychoeducation and cognitive behavioural therapy (CBT) have shown efficacy in schizophrenia [120] but have not specifically been assessed in PD psychosis. Active music therapy in PD patients showed benefits in behavioural, as well as in motor and affective functions [121]; however more systematic studies with a higher number of patients are required to study its potential effect as well as its long-term outcome.

### 4.2. Pharmacological Treatment

It is important to differentiate the treatment strategy of an acute and potentially life-threatening PD psychosis from a chronic setting.

The treatment strategy of acute psychotic episodes in PD is primarily to address and treat the underlying cause, including general measures, treatment of specific triggers, adaptation of medication, and/or addition of cholinesterase inhibitors in cognitively impaired PD patients (rivastigmine, donepezil, or galantamine) and antipsychotic agents such as clozapine or quetiapine when not manageable with the previous steps as shown in Table 5. If there is no response to neuroleptic agents, further investigations such as the measurement of amphetamines, methamphetamines, digoxin, T3, T4, TSH, and protoporphyrin should be considered. A transition from an acute to a chronic state can follow. In the chronic setting of a PD psychosis on the other hand, the first pharmacological approach is the optimization of the administered antiparkinsonian therapy, aiming at the lowest effective dose. The order in which the medication should be reduced is as follows: anticholinergic agents, selegiline, amantadine, dopamine receptor agonists, COMT-inhibitors, and lastly L-Dopa [124]. If reduction of medication however does not improve psychosis, the use of cholinesterase inhibitors or antipsychotic medication similarly as in the treatment strategy of the acute onset PD psychosis needs to be evaluated.

The main antipsychotic drugs used in PD psychosis are clozapine and quetiapine. Clozapine is slightly stronger than quetiapine but has a greater risk to induce agranulocytosis, with an estimated overall prevalence of 1-2% [125].

#### 4.2.1. Clozapine

Clozapine is an atypical antipsychotic whose mechanism of action is only partially understood, being thought to mainly act as an antagonist of dopamine D2 receptors and serotonin 2A receptors. It was first produced in 1958 and sold commercially after 1972. A double-blind, placebo-controlled study on clozapine for the treatment of drug-induced psychosis in PD (PSYCLOPS trial) showed an effectiveness of low-dose clozapine without worsening of motor function and response maintenance over at least 4 months in PD patients with psychosis [126]. A further double-blind, placebo-controlled study by Pollak et al. could find a statistically significant improvement in psychosis scores when compared to placebo, without significant motor function worsening, when using a low dose of clozapine of 50 mg/day. They also found wearing-off of the effect after discontinuation of the therapy [127]. These studies support the effectiveness of low-dose clozapine for the management of psychosis in PD.

#### 4.2.2. Other Antipsychotics

The use of newer antipsychotics was to follow the discovery of clozapine. The beneficial use of atypical antipsychotics in PD has been associated with a statistically significant increased risk of mortality, quetiapine being the weakest one associated with this fatal outcome, as studied in a recent trial by Weintraub et al. [128], so that studies to assess its efficacy in PD psychosis have been sought after.

Quetiapine is a dibenzothiazepine derivative structurally related to clozapine and was approved by the FDA in 1997 [129]. A study by Morgante et al. analyzed in a randomized, rater-blinded trial the effect of clozapine versus quetiapine in 45 PD patients with drug-induced psychosis over 12 weeks and concluded an equally efficacious potential of both drugs with unchanged motor scores among both groups [130]. A further study comparing the two medications by Merims et al. in 27 PD patients with recent-onset psychosis showed similar results but showed an overall trend of clozapine over quetiapine to control hallucinations (*P* = 0.097) and an advantage in reducing delusions (*P* = 0.011). Nonetheless the relatively high incidence of agranulocytosis under clozapine therapy poses a limitation in its use and the alternative of using quetiapine remains of importance [131]. Nonetheless, other studies comparing quetiapine to placebo in a total of 31 subjects [132], 58 subjects [133], and 24 subjects [134] failed to show efficacy on hallucinations when comparing quetiapine with placebo.

Studies on olanzapine, another atypical antipsychotic agent with dopamine D2 receptor and serotonin 2A receptor antagonism, could show no significant improvements in drug-induced PD psychosis and significant worsening in motor function in PD patients when compared to placebo in two placebo-controlled trials [135, 136].

Summarising the above-mentioned antipsychotic drugs as shown in Table 6, clozapine is efficacious for the treatment of psychosis in PD with an acceptable risk of side effects, if blood cell count is monitored. For quetiapine there is currently insufficient evidence to conclude on its efficacy for PD psychosis, but its safety profile shows benefit with no need of monitoring. For olanzapine, there is no evidence of efficacy in PD psychosis and an unacceptable motor function deterioration consequently making this compound not recommended for use in PD psychosis.

Less commonly used compounds such as risperidone have shown to be effective in the treatment of PD psychosis, nonetheless worsening motor function [138], so that it is currently not recommended as a treatment for PD psychosis. Ziprasidone has shown to improve PD psychosis symptoms in one small randomized single-blind parallel comparison study between ziprasidone and clozapine in 14 patients by Pintor et al., with no worsening of motor scores or cognitive function when comparing both intervention arms [139]. But to draw clinically relevant conclusions further studies with higher sample sizes are needed. Finally, melperone, another atypical antipsychotic of the butyrophenone chemical class, showed no benefit in the treatment of PD psychosis when compared to placebo in an unpublished double-blind, placebo-controlled trial by Friedman [140].

#### 4.2.3. Pimavanserin

Pimavanserin, a selective 5-HT2A inverse agonist without dopaminergic, adrenergic, histaminergic, or muscarinic effect [141], has been approved by the FDA [142] in September 2014 and has shown to be effective and safe in the treatment of PD psychosis, reducing hallucinations and delusions without affecting motor function [143, 144], which is a commonly observed drawback of most antipsychotic drugs due to dopamine antagonism. Recent studies have shown the potential of this drug to improve psychotic symptoms among PD patients [144, 145]. Results showed a significant improvement in measures of psychosis in PD patients without impairing motor function in one study by Meltzer et al. [145] and a statistically significant decrease in SAPS-PD scores in a bigger cohort of 199 PD patients in a randomized, double-blind, placebo-controlled phase 3 trial by Cummings et al. [144]. But pimavanserin and commonly used antipsychotics have up to now not been systematically compared.

#### 4.2.4. Cholinesterase Inhibitors

Clinical trials to assess the potential benefit of the widely used cholinesterase inhibitors for the treatment of dementia have been studied in PD psychosis due to its close relationship but have shown no beneficial effect on PD psychosis to date [73]. A randomized, placebo-controlled trial of donepezil in cognitive impaired PD patients showed a beneficial effect in memory, with no differences in psychiatric status or motor function [146] and an overall reduced tolerability with the recommendation of careful monitoring when used in PD patients. Another placebo-controlled study of rivastigmine in cognitive impaired PD patients showed moderate improvements in cognitive function but also higher rates of side effects, such as nausea, vomiting, and tremor [106]. Nonetheless, treatment of hallucinations in DLB has been reported to be efficacious with cholinesterase inhibitors such as donepezil [147, 148] and a clinical trial on the effect of donepezil in PD psychosis is currently under way [149]. Recommendation to use cholinesterase inhibitors in PD psychosis in cognitively impaired patients is currently supported by some authors [122, 123].

### 4.3. Electroconvulsive Therapy (ECT) and Deep Brain Stimulation (DBS)

Electroconvulsive therapy has shown beneficial effects in the treatment of neuropsychiatric symptoms in PD in some studies in the literature. The most recently published study on the effect of ECT in 29 drug-refractory PD patients with psychiatric symptoms, 12 of them having psychosis and depression and one having isolated psychosis, showed an improvement in measures of motor as well as nonmotor function assessed by means of different scales as seen in Table 7 [137].

This goes along other previous studies on ECT performed on PD patients with psychiatric comorbidities as summarised in Table 8, where most of the studies showed improvements in clinical impression and in the used scales. The mechanism of action of ECT is currently not known; potential postsynaptic dopamine receptor upregulation in the striatum, an increase in postsynaptic dopamine responsiveness, and an increase in levels of L-Dopa in the central nervous system by disrupting the blood-brain-barrier are thought to be underlying [150, 151]. As to its antipsychotic and antidepressant effect, enhancement of serotoninergic neurotransmission and mesocorticolimbic pathway activation has been postulated [152]; a PET study in PD patients with psychosis and depression showed that ECT led to an increase in metabolism in the anterior cingulate cortex and hippocampus, the latter of both showing correlation with a reduction of positive symptoms assessed by the Hamilton Depression Rating Scale (HDRS) [153]. Safety and tolerability issues have not been reported under ECT so far; nonetheless the performed studies to date are only small sample sized, have not been blinded, and have not assessed long-term efficacy. A large, sham-controlled study on the effect of ECT for the treatment of neuropsychiatric symptoms in PD is therefore highly needed. Studies on the effect of ECT in patients with deep brain stimulators have so far not been assessed; an undergoing collection of data to that purpose is under way [154].

Deep brain stimulation (DBS) has been reported as causing psychosis as a potential severe adverse event in PD patients [155], but the direct link to this complication is often overlapped by comorbid conditions and therefore contradictory in the actual literature [155, 156]. Its potential to treat psychiatric disorders in non-PD patients has furthermore shown some promising results in psychiatric conditions such as obsessive compulsive disorders [157]. Nonetheless, current evidence of DBS effect on PD psychosis is scarce and knowledge on its effect, whether improving or worsening the symptoms, cannot be stated at this point of time [19]. A clinical trial for the assessment of DBS for the treatment of resistant schizophrenia is currently under way; whether, if positive, the results could be potentially extrapolated to PD in the future remains open.

## 5. Conclusion

Although psychosis was thoroughly studied in recent years after being identified as one of the most relevant nonmotor features in PD, standardized guidelines for the management of PD psychosis are not available. Interestingly, psychosis, being one of the hallmarks of psychiatric illnesses such as schizophrenia with patients typically presenting with auditory features [21], presents rather differently in PD patients, where visual hallucinations clearly predominate, implicating a potentially different entity and thus underlying pathophysiological mechanism, further challenging the actual use of the same treatment options for both. In addition, current hypotheses on the underlying pathophysiological mechanisms including neurotransmitter dysregulation, structural/functional brain imaging abnormalities, and blood and CSF based biochemical measurements are sparse and inconsistent, supporting the lack of knowledge and the need of further investigation in order to potentially develop target oriented drugs in the nearer future. Currently undergoing studies on novel drugs for PD psychosis are expected to produce results in due time; ideas of further intervention strategies such as deep brain stimulation or stem cell therapy are being addressed for other causes of psychosis and could, if efficient, pose further options in the future. For now it remains clear that much more effort needs to be put into understanding this condition.

## Figures and Tables

**Box 1:**
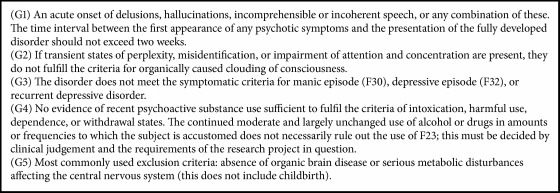
The ICD-10 classification of mental and behavioural disorders: definition criteria for acute and transient psychosis. F23, F30, F32: diagnosis codes of psychotic (F23) and mood disorders (F30 and F32) taken from ICD-10 guidelines; reference: taken from WHO International classifications, ICD-10 guidelines [15].

**Box 2:**
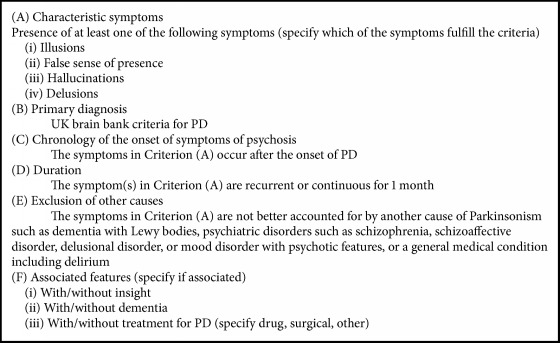
Proposed criteria for psychosis in Parkinson's disease. PD, Parkinson's disease; UK, United Kingdom; references: Ravina et al., 2007, and Fenelon et al., 2008 [22, 23].

**Table 1:** Studies on the prevalence of psychotic symptoms in PD.

Study	*N*	Setting/design	Assessment instruments	Main findings
Celesia et al., 1970	45	Outpatient/prospective longitudinal	Columbia disability scale	17.7% developed psychosis (delusions, hallucinations, behavioral disorder)
Sweet et al., 1976	100	Outpatient/retrospective	Cornell, weighted scale, WAIS	60% agitated confusion
Moskovitz et al., 1978	88	Outpatient/retrospective	No	48% experienced vivid dreams (30.7%), hallucinations (29.5%), illusions (5.7%), and nonconfusional (9.1%), confusional psychoses (3.4%)
de Smet et al., 1982	75	Inpatient/retrospective	No	31% confusional states
Tanner et al., 1983	775	Outpatient/retrospective	HY	33% hallucinations
Fischer et al., 1990	25	Inpatient/retrospective	HY, MMSE	80% at least one episode of “pharmacotoxic psychosis”
Sanchez-Ramos et al., 1996	214	Outpatient/prospective cross-sectional	HY, MMSE	25.7% visual hallucinations
Inzelberg et al., 1998	121	Outpatient/prospective cross-sectional	HY, SMT	29% visual, 8% visual and auditory hallucinations
Aarsland et al., 1999	245	Community/prospective cross-sectional	UPDRS, MMSE, DSM-III-R, MADRAS	25.5% vivid dreaming, 9.8% hallucinations with insight retained, and 6% severe hallucinations or delusions
Fenelon et al., 2000	216	Outpatient/prospective cross-sectional	UPDRS, HY, MMP, CES-D, DSM-IV	39.8% hallucinations. Minor hallucinations 25.5%, formed visual hallucinations 22.2%, and auditory hallucinations 9.7%
Giladi et al., 2000	172	Outpatient/prospective cross-sectional	HY, MMSE, DSM-IV, ADAS-cog	27% had psychosis
Goetz et al., 2001	60	Outpatient/prospective longitudinal	UPDRS, HY, RHI	Hallucinations increased from 33% at baseline to 44% at 18 months and 63% at 48 months
Holroyd et al., 2001	102	Outpatient/prospective cross-sectional	DSM-IV, TICS, GDS	29.4% had hallucinations or delusions
Doe De Maindreville, 2004		Outpatient/prospective longitudinal	UPDRS, HY, MMP, CES-D, DSM-IV	Hallucinations increased from 41.7% to 49.6% over 12 months

HY: Hoehn and Yahr staging; SMT: Short Mental Test; MMSE: Mini Mental State Examination; UPDRS: Unified PD Rating Scale; MMP: Mini Mental Parkinson; DSM-III-R: Diagnostic and Statistical Manual for Psychiatric Disorders, revised third edition; DSM-IV: Diagnostic and Statistical Manual of Mental Disorders, 4th edition; MADRAS: Montgomery and Asberg Depression Rating Scale; RHI: Rush Hallucination Inventory; ADAS-cog: Alzheimer's Disease Assessment Scale (ADAS) Cognitive Section; TICS: Telephone Interview for Cognitive Status; GDS: Geriatric Depression Scale; WAIS: Wechsler Adult Intelligence Scale; CES-D: Center for Epidemiologic Studies-Depression self-rating scale; reference: Papapetropoulos and Mash, 2005 [31].

**Table 2:** Recommended scales for the assessment of psychosis in Parkinson's disease.

Scale	Objective	References
PD nonmotor symptom scale	Risk of developing psychosis	Chaudhuri et al., 2007
MDS-UPDRS I, item 1.2	Presence and severity of psychosis	Goetz et al., 2007
Parkinson Psychosis Questionnaire (PPQ)	Presence and severity of psychosis	Sawada and Oeda, 2013
Scale for Evaluation of Neuropsychiatric Disorders in Parkinson's disease (SEND-PD)	Presence, severity of psychosis, and other neuropsychiatric symptoms	Rodriguez-Violante et al., 2014
Scale for Assessment of Positive Symptoms (SAPS)	Presence, severity, and impact of psychosis	Voss et al., 2013

PD, Parkinson's disease; MDS-UPDRS 1: Movement Disorder Society Unified Parkinson's Disease Rating Scale 1; SEND-PD: Scale for Evaluation of Neuropsychiatric Disorders in Parkinson's disease; SAPS: Scale for Assessment of Positive Symptoms; reference: Levin et al., 2016 [3].

**Table 3:** Features of delirium versus psychosis in PD.

Features	Delirium	Psychosis
Onset	Acute	Insidious
Course	Fluctuating, usually resolving over days to weeks	Progressive
Conscious level	Often impaired; can fluctuate rapidly; can be drowsy or hyperaroused	Clear
Cognitive defects	Poor short-term memory, poor attention span	Subtle
Hallucinations	Common, especially visual	Common especially complex visual or auditory
Key symptoms	Inattention, thought disorganisation, day-night reversal	Hallucinations, delusions, thought insertion, withdrawal or broadcast, passivity phenomena, phantom boarder
Medical status	Abnormal	Normal

Reference: Vardy et al., 2015 [58].

**Table 4:** Clinical and demographic features of PD patients with and PD patients without hallucinations after following up prospectively.

	PD-mH (*n* = 21)	PD-NH (*n* = 29)	*P*
Age, y	71.1 ± 7	65.8 ± 12	0.06 (*t*-test)
Education, y	8.4 ± 4	9.4 ± 5	0.45 (*t*-test)
Sex, ♂	57.10%	55.60%	0.91 (*χ*^2^)
Disease duration, months from onset of motor symptoms	22.8 ± 10	28.8 ± 14	0.12 (*t*-test)
UPDRS-III, at baseline	18.3 ± 9	20.1 ± 8	0.47 (*t*-test)
Hoehn & Yahr, at baseline	1.9 ± 0.2	2.1 ± 0.5	0.19 (*t*-test)
Predominance of motor symptoms, right%	52.3	65.5	0.23 (*χ*^2^)
Depression, %	52.3	41.40%	0.39 (*χ*^2^)
Anxiety, %	47.6	48.2	0.91 (*χ*^2^)
Apathy, %	42.8	55.1	0.47 (*χ*^2^)
Insomnia, %	38.1	41.3	0.68 (*χ*^2^)
Daytime sleepiness, %	38.1	27.5	0.45 (*χ*^2^)
RBD, %	38.1	10.3	0.03 (*χ*^2^)
Hyposmia, %	33.3	27.5	0.80 (*χ*^2^)

PD-mH: Parkinson's disease with minor hallucinations; PD-NH: PD without hallucinations; UPDRS-III: Unified PD Rating Scale, motor section; RBD: REM sleep behaviour disorder; reference: Pagonabarraga et al., 2016 [72].

**Table 5:** Proposed treatment strategies of acute, secondary psychosis in Parkinson's disease.

Step	Action	
I	General measures	Reestablishment of circadian rhythms
		Reestablishment of normal-level sensory inputs
		Hearing and vision aids
		Reestablishment of familial environment

II	Treatment of specific triggers	Treatment of infection, dehydration
		Balancing electrolytes, glucose, vitamins, hormones
		Treatment of heart insufficiency

III	Elimination of nonessential medication	Particularly anticholinergic, antiglutamatergic, sedating drugs

IV	Reduction of anti-Parkinson medication	Anticholinergics > amantadine > MAO-B-inhibitors > dopamine agonists > COMT-inhibitors > L-dopa retard > L-dopa nonretarded

V	Cholinesterase inhibitors in cognitively impaired patients	For example, rivastigmine 6–12 mg/d 2-3/d, or donepezil 5–10 mg/d 1/d (off-label), or galantamine 4–32 mg/d 2-3/d (off-label)

VI	Antipsychotic medication	Clozapine 12.5–62.5 mg/d (first-line), or quetiapine 12.5–75 mg/d (off-label)

COMT, catechol-O-methyltransferase; MAO-B, monoamine oxidase B; reference: Levin et al., 2016 [3], taken from Seppi et al., 2011 [122] and Connolly and Lang, 2014 [123].

**Table 6:** Summary of commonly used antipsychotic drugs to treat PD psychosis.

Drug	Efficacy	Safety	Practice Implications
Clozapine	Efficacious	Acceptable risk with specialized monitoring	Clinically useful
Olanzapine	Unlikely efficacious	Unacceptable risk	Not useful
Quetiapine	Insufficient evidence	Acceptable risk without specialized monitoring	Investigational

Reference: Seppi et al., 2011 [122].

**Table 7:** Clinical evaluation with scales before and after the sessions of electroconvulsive therapy (ECT).

Scales	Before ECT (mean ± SD)	After ECT (mean ± SD)	*P*
UPDRS III (*n* = 23)	45.2 ± 14.1	27.6 ± 11.9	<0.001
UPDRS IV (*n* = 23)	7 ± 3.1	2.7 ± 1.1	<0.001
HY (*n* = 21)	3.2 ± 0.6	2.1 ± 0.5	<0.001
MMSE (*n* = 27)	26.5 ± 3.9	26.3 ± 4.2	0.79
CGI (*n* = 27)	4.7 ± 0.8	2.9 ± 1	<0.001
BPRS (*n* = 27)	18.4 ± 8.6	9.6 ± 5.7	<0.001
HDRS (*n* = 27)	21.1 ± 2.2	10.5 ± 3	<0.001

ECT: electroconvulsive therapy; SD: standard deviation; UPDRS: Unified Parkinson's Disease Rating Scale; HY: Hoehn and Yahr; MMSE: Mini Mental Status Examination; CGI: clinical global impression; BPRS: Brief Psychiatric Rating Scale; HDRS: Hamilton Depression Rating Scale; reference: Calderón-Fajardo et al., 2015 [137].

**Table 8:** Summary of published reports of electroconvulsive therapy for the management of neuropsychiatric symptoms in subjects with Parkinson's disease.

Study	Sample	Neuropsychiatric disorder	Measurements	Findings
Nishioka et al., 2014	4	Psychosis	NPI HAM-D	Improvement of 89.8% in the NPI
				Improvement of 81.1% in the HAM-D
Sadananda et al., 2013	1	Psychosis	PANNS	Improvement of 77.3% in the PANNS
Muhammad et al., 2012	1	Obsessive-compulsive disorder	Clinical impression	Improvement
Usui et al., 2011	8	Psychosis	SAPS	Improvement of 65.8% in the mean SAPS total
Ducharme et al., 2011	1	Depression	Clinical impression	Improvement
Ueda et al. 2010	5	Psychosis	BPRS HAM-D	Improvement of 89.2% in the BPRS
				Improvement of 83.8% in the HAM-D
Bailine et al. 2008	1	Psychotic depression	Clinical impression	Improvement
Lance et al., 1998	1	Depression	Clinical impression	Improvement
Mollentine et al., 1998	25	Depression and/or psychosis, dementia	BPRS HAM-D	
Nymeyer et al., 1997	1	Depression	Clinical impression	Improvement
Factor et al., 1995	2	Depression and/or psychosis	Clinical impression	Improvement
Sandky et al., 1993	1	Psychotic depression	Clinical impression	Modest improvement
Oh et al., 1992	11	Depression and/or psychosis	Clinical impression	82% of the patients improved
Zwil et al., 1992	8	Depression and/or psychosis		
Friedman et al., 1992	5	Depression and/or psychosis	Clinical impression	Improvement
Stern, 1991	1	Depression	Clinical impression	Improvement
Liberzon et al., 1990	1	Psychotic depression	Clinical impression	Improvement

NPI: neuropsychiatric inventory; HAM-D: Hamilton Depression Rating Scale; PANNS: Positive and Negative Syndrome Scale; SAPS: Scale for the Assessment of Positive Symptoms; BPRS: Brief Psychiatric Rating Scale; reference: Calderón-Fajardo et al., 2015 [137].
